# Employing Expression-Matched Controls Enables High-Confidence Proximity-Based Interactome Classification

**DOI:** 10.1016/j.mcpro.2025.101001

**Published:** 2025-05-27

**Authors:** Fulin Jiang, Xuezhen Ge, Eric J. Bennett

**Affiliations:** School of Biological Sciences, Department of Cell and Developmental Biology, University of California, San Diego, La Jolla, California, USA

## Abstract

Proximity labeling approaches have been widely utilized to define protein interactomes. Due to the inherent promiscuity of proximity labeling using TurboID-based approaches, identification and adoption of appropriate labeling controls is a pivotal step to mitigate background interference and enhance interactome assignment accuracy. Here, we evaluate the effectiveness of both expression controls and data normalization strategies in generating high-confidence interactome maps. We demonstrate that the extent of control of TurboID protein expression is strongly correlated with overall signal intensity and the number of identified proteins from streptavidin-enrichments. Discordant expression levels between the bait and control samples result in high-frequency false-negative and false-positive identifications. Data normalization strategies help correct these expression differences but also introduce data distortion for proteins with high or low endogenous expression. Using the ubiquitin ligases RNF10 and HUWE1 as bait proteins, we demonstrate that matching TurboID expression between control and bait proteins allows for similar sampling of non-specific interactions. Using a matched expression strategy results in significantly reduced background interference and increases the accuracy of interactome assignments. These results document the need to alter proximity-labeling experimental workflows to include the generation of matched expression controls to enhance proximity labeling proteomics interactome mapping robustness and reproducibility.

## Highlights

• Background proteins arise from bead adsorption and self-labeling by TurboID.
• TurboID or TurboID-GFP is suitable as a general control for proximity labeling.
• TurboID expression closely correlates with enriched protein quantity and intensity.
• TurboID-matched controls reduce background interference in differential analysis.

## Graphical Abstract

**Figure:**
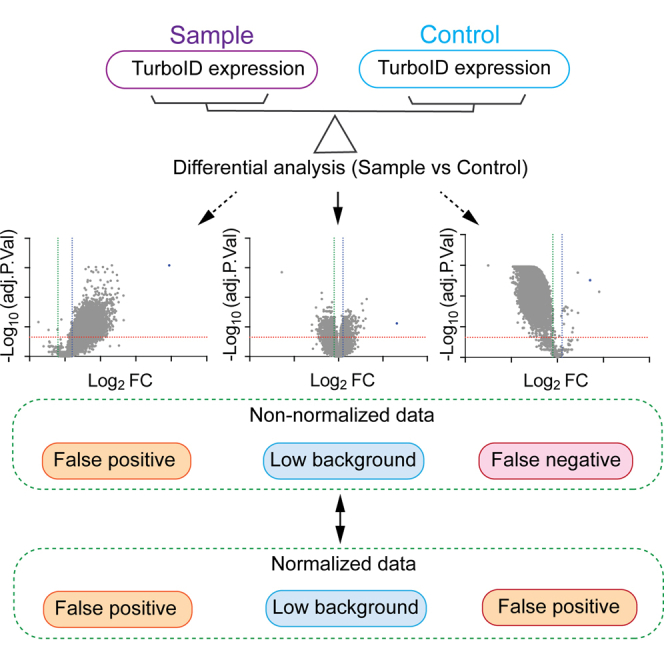

## In Brief

Proximity labeling is a powerful tool for mapping protein interactomes. This study evaluates how selection of expression matched controls and data normalization strategies affect the accuracy of interactome assignment using proximity labeling approaches. Using RNF10 and HUWE1 as baits, we show that expression-matched controls reduce background interference and improve interactome assignment, leading to an updated TurboID workflow with enhanced robustness and reproducibility.

Mapping protein interactions provides crucial insights into cellular organization and function. Proximity labeling has emerged as a powerful approach that employs enzyme-catalyzed tagging to identify proteins in close spatial proximity to a bait protein. Coupled with advanced proteomics, this technique enables comprehensive mapping of protein-protein interactions, including both direct and indirect associations and provides spatial context by capturing the local proteome surrounding the bait (1, 2). The proximity labeling approach has gained widespread adoption due to its efficiency, minimal disruption to living cells, and ability to capture transient interactors and low-abundance proteins (1, 3, 4).

Enzymes employed as proximity labeling reagents have undergone continuous refinement as these enzymes are crucial components of the proximity-labeling process. TurboID, an engineered biotin ligase derived from wild-type *Escherichia coli* BirA (5), was optimized *via* directed evolution approaches (3). Compared to its earlier version, BioID (6), TurboID exhibits significantly higher activity, which reduces labeling time from 18 to 24 h for BioID-based experiments to just 10 min. Additionally, TurboID combines the high labeling efficiency of ascorbate peroxidase (APEX)-based proximity labeling systems (7), which rely on exposing cells to toxic levels of hydrogen peroxide, with the non-toxic nature of the BirA enzyme family. Therefore, TurboID has been widely utilized for proximity labeling (PL) proteomics approaches.

Background interference, such as the nonspecific capture and identification of background proteins, is a significant challenge in proximity labeling proteomics. Selection and utilization of suitable controls are required to successfully monitor background interference and flag false-positive identifications. The controls commonly employed in TurboID PL proteomics can be categorized into five types (1): no TurboID expression (8, 9), (2) Untagged TurboID expression or TurboID expression as a fusion protein (*e.g.* TurboID-GFP) (10, 11), (3) TurboID fused to the bait protein without biotin supplementation (12, 13), (4) TurboID fused to specific mutants of the bait protein (14, 15), and (5) different experimental treatment conditions (*e.g.* comparing untreated to pathway perturbation) (16, 17). Among these, the first three can be regarded as general-purpose controls. The number of proteins identified using these controls can range from tens to thousands, depending on experimental conditions and mass spectrometer sensitivity. Consequently, the choice of controls can significantly impact differential analysis results and subsequent identification of the bait protein interactome. Undersampling background interference will greatly increase false positive identifications, whereas oversampling the background proteome can lead to exclusion of bona fide interactors (*i.e.* false negatives). To reduce background interference, several strategies have been reported (18, 19). These include simpler approaches, such as using more stringent washing conditions during affinity enrichment steps, as well as more sophisticated methods, such as expressing untagged biotin ligase to capture background labeling. Recent studies have introduced a T2A split/link design in which free TurboID was used as a control and TurboID translation is coupled to target protein translation (20). Despite improvements in using appropriate PL controls, many reported PL studies lack sufficient controls to determine reliable bait protein interactomes. Furthermore, there has not been a systematic evaluation of the characteristics of different types of controls that should be considered when developing experimental PL strategies.

Bait protein expression level is a widely recognized variable that significantly contributes to resulting protein interaction maps. In PL approaches, varying expressions of TurboID-bait protein fusions can dramatically alter the extent of overall protein biotinylation. These observations suggest that appropriate controls may need to be expressed at similar levels compared to the bait protein to ensure proper sampling of background labeling. However, it remains unclear if matching TurboID expression levels in controls is a critical component of PL experimental design. The practice of matching protein expression levels between controls and samples has been underutilized and insufficiently validated. Proteomics data normalization methods are often used to correct for biases arising from variations in injection volume, instrumental analysis, and sample processing (21). Theoretically, these normalization methods can adjust the intensities of captured proteins based on the TurboID-bait protein expression level to allow for direct comparison to control samples (22). Whether proteomics data processing steps can adequately correct for these expression differences remains an open question.

In this study, we examine the proteomic characteristics of three general-purpose controls and highlight the advantages of using TurboID-eGFP as a necessary negative control for PL approaches. We then demonstrate that the TurboID expression level significantly influences the number and signal intensity of identified background proteins. When the TurboID expression in the control does not align with that in the TurboID-bait protein fusion group, excessively high or low expression can lead to false-positive or false-negative results in differential analysis. An in-depth analysis of the characteristics of enriched proteins reveals that proteins with different endogenous expression levels display distinct enrichment patterns as TurboID expression changes, posing challenges for data normalization approaches. When TurboID expression in the control is significantly higher than in the sample, normalization introduces noticeable distortions. High-abundance proteins in the control group are suppressed, while low-abundance proteins are artificially elevated. High expression of the TurboID control enriches more proteins, and total intensity-based normalization strategies inevitably compress the quantification of known background proteins. This reduction in background protein intensities ultimately increases false-positive identifications in differential analyses. Our results illustrate the importance of using expression-matched TurboID-tagged controls and samples to minimize false positive identifications. We outline an optimized TurboID application workflow that incorporates steps to assist with matching expression levels between controls and samples. This improvement provides a robust theoretical and practical foundation for the rational selection and optimization of controls that can be employed in proximity labeling proteomics approaches to more precisely identify true positive interacting proteins.

## Experimental Procedures

### Experimental Design and Statistical Rationale

For all mass spectrometry experiments, the sample size was n = 3 biological replicates. The rationale is that n = 3 is a commonly used number of experimental replicates for proteomics experiments based on cell culture-based experiments and, when combined with label-free quantification, provides sufficient statistical power to detect significant but small differences with high accuracy. The control and sample groups were processed under identical conditions except for the factor under investigation. Detailed information on all experiments, including sample types, sample preparation, and data acquisition, is provided in supplemental Table S1. In total, six datasets were obtained, with each dataset corresponding to the same batch MS analysis. All experimental replicates and data points were included in the final analysis (supplemental Table S2). Proteins identified by only a single unique peptide were excluded to ensure data reliability, except for the protein of interest, UBE2D2/3, for which annotated spectra are provided (supplemental Table S2). Statistical methods are explained in more detail in the “MS data analysis and bioinformatics analysis” paragraph.

### Cell Culture

HEK293T and 293Flp-In cells were grown in Dulbecco’s modified Eagle’s medium (DMEM) containing 10% fetal bovine serum, 100 U/ml penicillin, and 100 U/ml streptomycin. HAP1 cells were cultured in Iscove’s Modified Dulbecco’s Medium (IMDM) supplemented with 10% fetal bovine serum, 100 U/ml penicillin, and 100 U/ml streptomycin. All cells were incubated at 37 °C with 5% CO_2_.

### Transfections

Plasmids were validated by restriction enzyme digestion followed by agarose gel electrophoresis to check the digestion fragments and subsequently confirmed by sequencing. Their expression after transfection was assessed *via* immunoblotting.

The Flp-In system (Thermo Fisher), through single locus integration and hygromycin selection, was used to generate stable doxycycline-inducible cell lines expressing V5-tagged proteins. Flp-In 293 cells were transfected with Flp-In expression vectors for TurboID-RNF10 using TransIT 293 transfection reagent (Mirus) according to manufacturer guidelines. Cells were seeded at 60% confluency, transfected for 24 h, followed by selection of stable expression clones with 2 μg/ml hygromycin. Treatment with 1μg/ml doxycycline for 24 h before harvesting was used to induce protein expression.

Lentiviral transduction was employed to generate stable cell lines expressing Flag-V5-tagged TurboID-RNF10. Cells were transfected using Mirus TransIT 293 transfection reagent with a combination of five helper plasmids: pHAGE-GAG-POL, pHAGE-VSVG, pHAGE-tat1b, pHAGE-rev, and pHAGE-V5-TurboID-RNF10. After 24 h, the media were replaced with fresh media. The viral supernatant was collected, filtered through a 0.45 μm sterile syringe filter, and combined with 2 μl of 6 mg/ml polybrene. The resulting viral mixture was added to cells that were at approximately 50% confluency and allowed to infect for 24 h. Stable clones were then selected using 1 μg/ml Puromycin.

For piggyBac-based transfection, HAP1 or 293T cells were seeded at 60% confluency 1 day before transfection. The cells were then co-transfected with a donor plasmid containing the gene of interest flanked by piggyBac terminal repeat sequences and a helper plasmid (pb210) expressing the piggyBac transposase, using a Lipofectamine 3000 or 2000 transfection reagent according to the manufacturer’s instructions. After 24 h, the medium was replaced with fresh medium. Stable clones are selected 48 h post-transfection using 1 μg/ml Puromycin. Doxycycline (1 μg/ml) is added 1 day before selection, and selection continues for 1 week, with medium changes every 2 to 3 days.

### TurboID Knockin

TurboID was knocked in 293FlpIn cells by the CRIS-PITCh (v2) system (23, 24). The selected guideRNA is “ggggagctcagcggcatcaa". Two solutions were prepared: three plasmids, pX330S-2-PITCh, CRISPR-Cas9 vector, and CRIS-PITCh (v2) vector, were added to 150 μl of Opti-MEM, and 3 μl of Lipofectamine 2000 was added to another microtube containing 150 μl of Opti-MEM. The two solutions were then mixed and incubated for 15 min at room temperature before the mixture was added to the cultured cells, which were incubated in a CO_2_ incubator. Cells were transferred to 10 cm plate, and 72 h post-transfection, the medium was replaced daily with 10 ml of DMEM containing 1 μg/ml puromycin for 14 days. After selection, the cells were cultured until colonies had grown sufficiently for knock-in confirmation. A portion of cells from each colony was collected, and genomic DNA was extracted. PCR was performed using KOD polymerase with primers specifically designed to amplify the 5′ and 3′ junctions of the knock-in cassette separately. The PCR products were run on a 2% (wt/vol) agarose gel, and the desired bands were excised. The gel fragments were collected into microtubes, and the DNA was purified using the QIAquick Gel Extraction Kit. The genotype was confirmed by sequencing. The primers “GGAAAAATGTCGCCATGAAG” and “AAGAGTTCTTGCAGCTCGGT” were used to check the 5′ end junction of the inserted fragment. The primers “CAGGACGGAGTTATCAAACCC” and “TTACCGCTCTTGGGTTTAGACT” were used to check the 3′ end junction of the inserted fragment.

### Flow Cytometry Analysis

To evaluate GFP levels at cell surface, the cells were collected and washed with PBS, cells were subjected to flow cytometry detection on a BD LSRFortessa flow cytometer. The mean fluorescence intensity of GFP was analyzed using FlowJo v10 Software.

### Immunoblotting

Cell pellets were resuspended in a urea-based denaturing lysis buffer (8M urea, 50 mM Tris-HCl pH 8.0, 75 mM NaCl, 1 mM Na_3_VO_4_, 1 mM NaF, 1 mM β-glycerophosphate, 40 mM NEM) supplemented with an EDTA-free protease inhibitor cocktail and kept on ice during the preparation. The cell lysates were sonicated for 10 s at an output of 8 mW using a Fisher Scientific membrane dismembrator model 100 equipped with a microtip probe, then centrifuged at 15,000 rpm for 10 min at 4 °C. Protein concentrations were determined using a BCA Protein Assay (Thermo Scientific Pierce, 23225). Laemmli sample buffer containing β-mercaptoethanol was added to the lysates, which were then heated at 95 °C for 10 min. After cooling to room temperature and a brief centrifugation, lysates were resolved on 10% or 12% Tris-glycine SDS-PAGE gels and transferred to PVDF membranes (BioRad, 1620177) using Bjerrum semi-dry transfer buffer (48 mM Tris base, 39 mM glycine, 0.0375% SDS, 20% methanol, pH 9.2) and a Bio-Rad Turbo Transfer apparatus at 25V for 30 min. Membranes were blocked with 5% nonfat dry milk in TBST for 1 h, followed by overnight incubation with primary antibodies diluted in 5% BSA. Blots were developed using Clarity Western ECL Substrate (BioRad, 1705061) and visualized on a Bio-Rad Chemi-Doc XRS+ system. All immunoblots were analyzed using ImageLab software (BioRad).

### Biotin Depletion From Culture Medium

To minimize background biotinylation caused by TurboID, biotin-depleted fetal bovine serum was prepared. 120 μl of streptavidin agarose beads were added to 50 ml of dialyzed FBS and incubated overnight at 4 °C with rotation. The supernatant was collected and used to prepare complete culture medium. After medium preparation, the entire medium was filtered through a 0.22 μm membrane to remove any remaining beads or particulate matter. This biotin-depleted medium was used for TurboID-related experiments.

### Proximity Labeling

Cells were plated into 6-well plates with 2 ml of medium containing doxycycline (DOX) to achieve 70 to 80% confluency after 24 h. For non-inducible systems, puromycin was not added to the culture medium. To initiate labeling, the medium in each well was replaced with warm, freshly prepared biotin-containing complete DMEM at a final concentration of 100 μM. Cells were incubated with biotin at 37 °C for 60 min. The labeling reaction was stopped by placing the cells on ice and gently washing the samples five times with 1 ml of ice-cold DPBS. Cells were detached using 1 ml of ice-cold DPBS per well through pipetting, and the suspension was collected into microcentrifuge tubes. For strongly adherent cells, a cell scraper was used. The cells were pelleted by centrifugation at 300 g at 4 °C for 3 min, the supernatant was removed, and the cell pellets were stored at −80 °C.

### Whole Cell Proteomics Sample Preparation

Cell suspensions were centrifuged at 300 g for 3 min at room temperature, washed with phosphate-buffered saline (PBS), and pelleted before being resuspended in lysis buffer (8 M urea, 50 mM Tris-HCl pH 8.0, 75 mM NaCl, 1 mM Na_3_VO_4_, 1 mM NaF, 1 mM β-glycerophosphate). The lysate was centrifuged at 15,000*g* for 10 min at 4 °C, and the supernatant was collected. Protein yield was determined using Thermo Pierce BCA (bicinchoninic acid) protein assays, following the manufacturer’s protocol. For each sample, 20 μg of protein from the cell lysate was used. Samples were treated with 5 mM tris (2-carboxyethyl)-phosphine (TCEP) and incubated at room temperature for 30 min. Subsequently, 10 mM iodoacetamide was added, and the samples were incubated in the dark for 45 min at room temperature. Iodoacetamide was quenched with 15 mM dithiothreitol (DTT) for 10 min. The urea concentration was diluted to 1 M using 50 mM Tris-HCl, and proteins were digested by adding trypsin (1:50 enzyme-to-protein ratio, wt/wt) and LysC (1:100 enzyme-to-protein ratio, wt/wt). Digestion was carried out overnight at 37 °C with shaking at 1000 rpm. The resulting peptides were desalted using StageTips packed with C18 material. Peptides were loaded onto the StageTips, washed with 0.1% formic acid, and eluted with 80% acetonitrile in 0.1% formic acid. The cleaned peptides were dried under vacuum and reconstituted in 5% formic acid with 5% acetonitrile. Samples were stored at −20 °C until LC-MS/MS analysis.

### Proximity Labeling Proteomics Sample Preparation

Lysis buffer consisted of 50 mM Tris (pH 8.0), 150 mM NaCl, 0.5% NP-40, phosphatase inhibitors (1 mM NaF, 1 mM β-glycerophosphate, and 1 mM sodium orthovanadate), and Roche protease inhibitor tablets. The sample was incubated on ice for 5 min before sonication and centrifugation. Protein concentration was determined using a BCA assay, and 50 μg of total protein was used for streptavidin affinity enrichment. For each sample, 30 μl of a 50% slurry of streptavidin-agarose beads was used. The samples were incubated for 2 h at 4 °C on a rotating incubator. Following incubation, the supernatant was removed after centrifugation, and the beads were washed three times with wash buffer (50 mM Tris (pH 8.0), 150 mM NaCl, 0.1% NP-40) and three times with PBS. The samples were then subjected to reduction and alkylation. Digestion and subsequent desalting were performed using the same steps as described for whole-cell proteomics.

### LC-MS Analysis

Nanoflow reversed-phase chromatography was performed on nanoElute liquid chromatography system (Bruker Daltonics). Peptides were separated in 60 min at a flow rate of 400 nl/min on a commercially available reversed-phase C18 column (25 cm × 150 μm, 1.6 μm, IonOpticks). Mobile phases A and B were 0.1 vol% formic acid in water and 0.1 vol% formic acid in ACN, respectively. The fraction of B was linearly increased from 2% to 25% in 50 min, followed by an increase to 35% in 10 min and a further increase to 80% in 10 min before re-equilibration.

MS was performed with a hybrid TIMS quadrupole TOF mass spectrometer (Bruker timsTOF Pro2) *via* a CaptiveSpray nano-electrospray ion source in positive ion mode using data-independent acquisition. To achieve high data completeness and extensive proteome coverage, we utilize variable isolation windows (supplemental Table S3) that are strategically placed based on the density of precursors in the m/z and ion mobility (IM) planes based on reported methods (25). The IM range was set between 1.6 and 0.6 V/cm^2^. Accumulation and ramp times were fixed at 100 ms for all experiments. Consequently, each MS1 and MS2/dia-PASEF scan lasted 100 ms, plus additional transfer time, resulting in a cycle time of 2.6 s for a dia-PASEF method with 25 scans. The collision energy was adjusted based on the IM, decreasing from 59 eV at 1/K_0_ = 1.6 V/cm^2^ to 20 eV at 1/K_0_ = 0.6 V/cm^2^. The IM dimension was calibrated using three Agilent ESI Tuning Mix ions with m/z and 1/K_0_ values of 622.02, 0.98 V/cm^2^; 922.01, 1.19 V/cm^2^; and 1221.99, 1.38 V/cm^2^.

### MS Data Analysis and Bioinformatics Analysis

We utilized DIA-NN (1.9.2) to convert raw data into precursor and fragment identifications based on their 3D peak positions, including retention time (RT), m/z precursor, and ion mobility (IM) (26). All data were searched against the reviewed human proteome (UniProt, November 2021, 20,555 entries, excluding isoforms) using trypsin/LysC as digestion enzymes. Cysteine carbamidomethylation was set as a fixed modification, while methionine oxidation and methionine excision at the N-terminus were selected as variable modifications. Up to two missed cleavages and two variable modifications were allowed. Default settings were used, with modifications to the charge state (2–4). The precursor m/z range was restricted from 300 to 1200 for proteome analysis. The fragment m/z range was set from 100 to 1700, and mass accuracy for MS1 was set to 15 ppm. “Match between runs” was enabled, while “protein inference” was disabled. The quantification strategy used “robust LC (high precision).” Proteomics outputs were filtered to ensure a maximum q value of 1% at both precursor and global protein levels.

Protein intensities were calculated based on the precursor intensity matrix exported from DIA-NN using multiple strategies. Non-normalized approaches included (1): summing all precursor intensities for each protein (2), selecting the most intense precursor (Top1), and (3) applying the xTop method (27). Normalized approaches included (1): summing all precursor intensities and normalizing by the total precursor intensity per sample (2), summing DIA-NN RT-normalized precursor intensities, and (3) applying MaxLFQ (28). Unless otherwise stated, the total ion intensity normalization method was used in the primary analysis using R (version 4.5.0) in RStudio. Specifically, for each sample, the intensities of all peptide precursors were summed to obtain the total precursor intensity. Individual precursor intensities were then scaled by the ratio of 10,000,000 to the sample’s total precursor intensity.

Differential expression analysis was performed using the limma package (version 3.56.2) in R. Missing values were imputed using the 1% quantile approach, and expression data were log_2_-transformed before analysis. A design matrix was specified, and linear modeling was applied using lmFit, followed by contrast estimation (makeContrasts) and empirical Bayes moderation (eBayes). Features with an adjusted *p*-value (FDR <0.05) were considered significant. ROC curve analysis was performed using RNF10 interaction partners (supplemental Table S4) collected from BioGRID, STRING, and IntAct to benchmark differential analysis performance. Proteins were scored using the π-value, defined as the negative log10-transformed *p*-value multiplied by the sign of the log2 fold change (29). All the mass spectrometry raw data and associated analysis files generated in this study have been deposited in the ProteomeXchange Consortium *via* the PRIDE partner repository. Further details are provided in the Data Availability section.

All proteomics assays were performed in triplicate (n = 3) as biologically distinct samples. The data were analyzed using Prism 10.0 software (GraphPad) and are presented as the mean values (standard error of the mean, ± SEMs). Linear fitting of the data was conducted using Prism 10.0. Figure organization and editing were performed using Adobe Illustrator 2024.

## Results

### Evaluation and Characterization of Different TurboID Controls

**Fig. 1:**
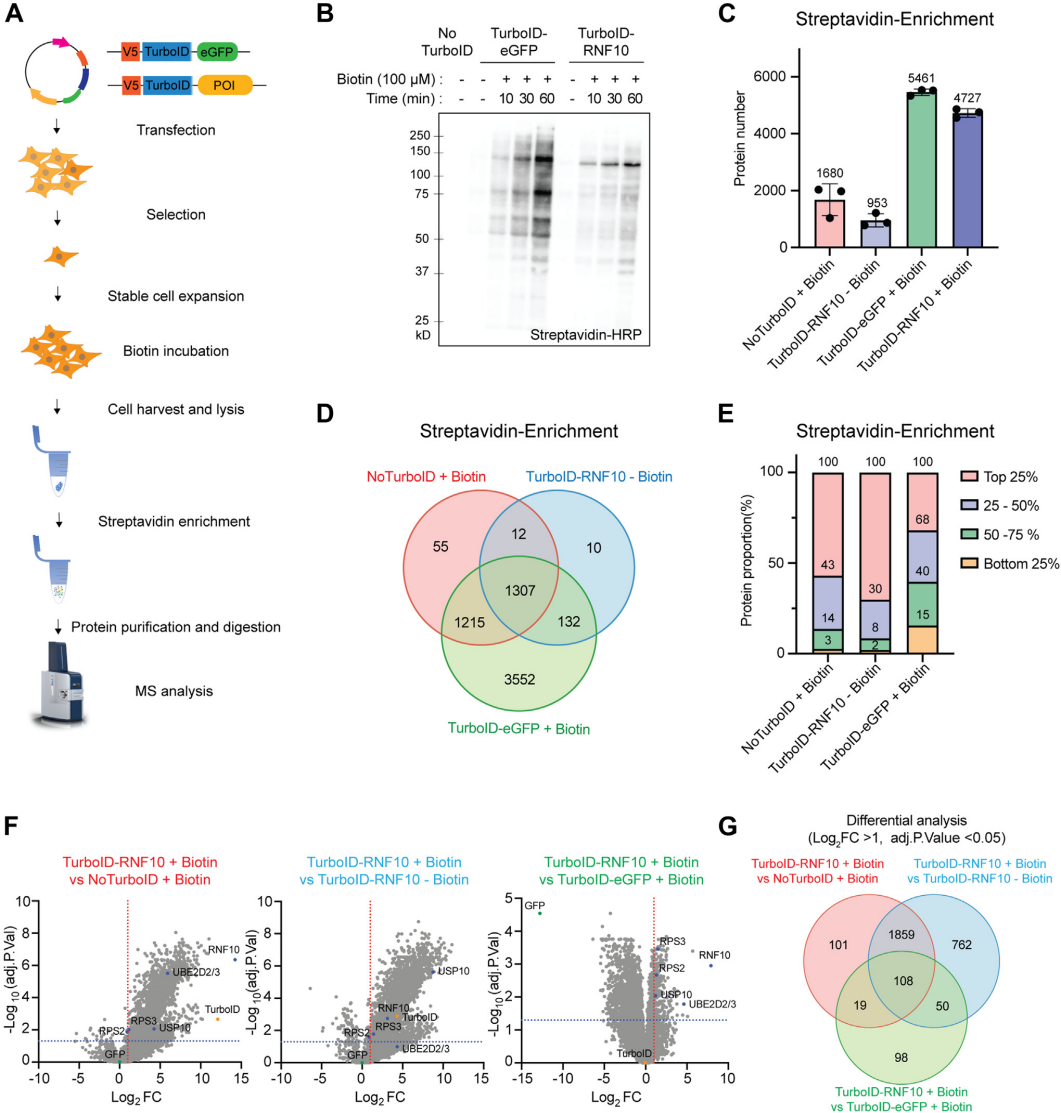
**Comparison of general-purpose TurboID controls**. *A*, schematic diagram of TurboID experimental workflow. *B*, HEK293 FlpIn cells with indicated TurboID expression were treated with or without doxycycline for 24 h. Whole cell extracts were analyzed by SDS-PAGE and total protein biotinylation was detected using streptavidin-HRP. Cells were supplemented with 100 μM biotin for the indicated times. *C*, the total number of identified proteins from streptavidin-based enrichments of whole cell extracts from cell lines as indicated. (n = 3). *D*, Venn diagram of depicting the overlap of identified proteins from streptavidin-enrichments using the indicated control groups. *E*, abundance distribution of identified proteins from streptavidin-enrichments using the indicated control groups. (n = 3). *F*, Volcano plot obtained from differential analysis using three types of controls. *G*, Venn diagram depicting the overlap of identified RNF10 interacting proteins using the indicated control groups. Significantly enriched proteins were defined using thresholds of a fold change greater than two and an adjusted *p*-value less than 0.05.

To begin to evaluate the effectiveness of different control groups when performing proximity-labeled proteomics experiments, we generated 293 Flp-IN cells with inducible expression of TurboID-tagged eGFP as a control group. Similar cell lines with inducible expression of TurboID-tagged RNF10 were generated to serve as the bait protein of interest (Fig. 1*A*). RNF10 is a ubiquitin ligase that ubiquitylates the 40S ribosomal proteins uS3 (RPS3) and uS5 (RPS2) when 40S progression during translation initiation becomes stalled (30). These ubiquitylation events destroy the entire 40S subunit. RNF10 was chosen as the test protein because there are few known interacting proteins for RNF10, and the few known interacting proteins are highly abundant ribosomal proteins that are regularly identified in control samples from many proteomics experiments. We confirmed the dox-inducible TurboID-eGFP and TurboID-RNF10 expression (supplemental Fig. S1, *A*–*C*). Overexpression of the TurboID-tagged WT RNF10, but not a mutant version that lacks ubiquitin ligase activity (CS) resulted in uS3 and uS5 ubiquitylation, confirming that TurboID-tagged RNF10 maintains interaction with its known substrates (supplemental Fig. S1, *D* and *E*). We then optimized biotin incubation time and concentration for the TurboID-eGFP and TurboID-RNF10 expressing cell lines (Fig. 1*B* and supplemental Fig. S1, *F* and *G*). Within the tested range, incubation time had a more significant impact on labeling efficiency than biotin concentration. For all subsequent experiments, cells were incubated with 100 μM biotin for 1 h.

We first evaluated the efficacy of three commonly used controls for proximity-labeling experiments: Parental 293Flp-IN cells without TurboID expression, TurboID-RNF10 expressing cells without biotin supplementation, and TurboID-eGFP expressing cells supplemented with biotin. Biotinylated proteins were enriched by streptavidin pulldown and identified by mass spectrometry. More than 1600 proteins were identified from cells without TurboID expression demonstrating both the sensitivity of the mass spectrometer, and the inherent high degree of nonspecific background binding to streptavidin agarose (Fig. 1*C*). Expression of either TurboID-tagged eGFP or RNF10 greatly increased the number of identified proteins with TurboID-eGFP expression resulting in the highest number of identified proteins highlighting the promiscuous nature of proximity-labeling using TurboID (Fig. 1*C*).

The overall profile of identified background proteins from the no-TurboID expression and the control lacking biotin supplementation was similar (Fig. 1*D*). These background proteins had two characteristics. First, they comprised mostly high-abundance proteins with over 50% of identified proteins coming from the top quartile of protein expression as determined by proteomic analysis of unfractionated whole-cell extracts (Fig. 1*E*). Second, the background proteins had higher than average isoelectric points, which may enhance interaction with streptavidin (supplemental Fig. S1*H*).

In contrast, the TurboID-eGFP group enriched for proteins across the abundance distribution (Fig. 1*E*) and exhibited isoelectric point values that were slightly higher than those of proteins identified in the other control groups (supplemental Fig. S1*H*). Proteins enriched in TurboID-eGFP group included most of the proteins enriched in the other two controls and had its own unique set of 3552 proteins (Fig. 1*D*). When TurboID-eGFP was used as a control for differential analysis, the background interference was significantly reduced compared to that from the other two controls (Fig. 1, *F* and *G* and supplemental Fig. S1*I*). This further demonstrates the advantages of using a TurboID-eGFP expression control for proximity labeling experiments.

### TurboID Expression Significantly Alters Background Labeling

**Fig. 2:**
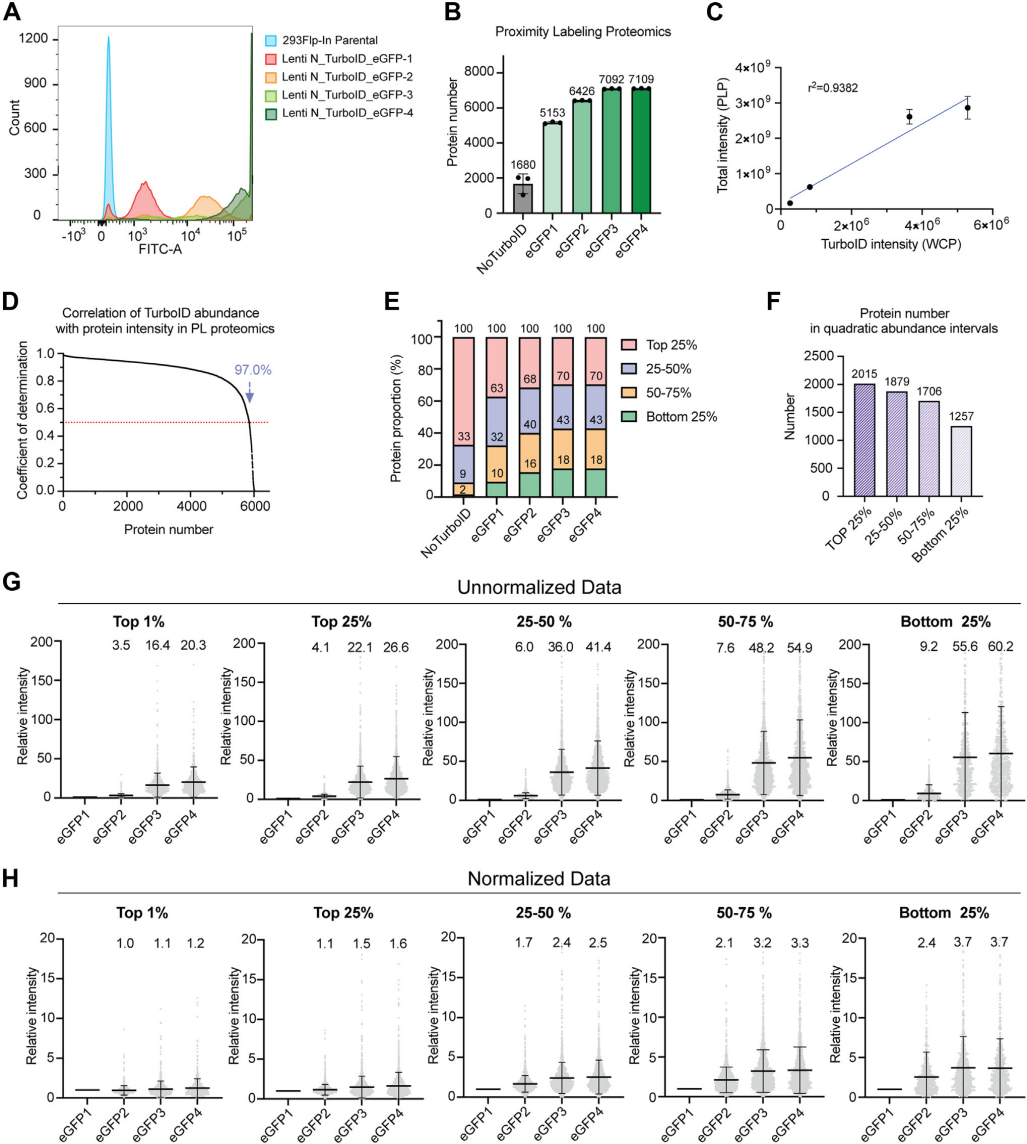
**Varying TurboID-eGFP expression controls**. *A*, distribution of GFP intensities from the indicated cell lines. *B*, total number of identified proteins from streptavidin-enrichments from the indicated cell line. *C*, correlation between TurboID expression obtained from whole-cell proteomics and total ion signal intensity from streptavidin-enrichments for the four TurboID-eGFP groups. *D*, correlation between TurboID expression obtained from whole-cell proteomics and the intensity of individual proteins identified from streptavidin-enrichments from the four TurboID-eGFP groups. The intensity of each protein across the four groups was linearly fitted with the TurboID expression values of each group to calculate the coefficient of determination (R^2^). Proteins with valid intensities in at least three groups were included in the analysis. *E*, abundance distribution of identified proteins from streptavidin-enrichments using the indicated control groups. *F*, number of proteins in different abundance quartiles from streptavidin-enrichments by combining the four TurboID-eGFP groups. *G* and *H*, relative intensity of enriched proteins from different abundance groups as compared to the intensity determined in the GFP-1 group. The number above each group indicates the mean relative intensity. Unnormalized (*G*) and normalized (*H*) data are shown for comparison.

Based on our results, we focused on optimizing the use of TurboID-eGFP as a control for proximity-labeling proteomics approaches. How varying TurboID expression levels alter proteome biotinylation and subsequent protein enrichment and quantification patterns remains unclear. To examine the effect of TurboID-eGFP expression on identified non-specific proteome labeling, we sorted TurboID-eGFP expressing cells into four different expression groups based on eGFP fluorescence intensity (Fig. 2*A*). We then used these cells with varying TurboID-eGFP expression to label and identify the eGFP interactome. Biotin incubation time was maintained at 60 min for all experiments. The number of identified proteins increased with increasing TurboID expression (Fig. 2*B*), and the total signal intensity of all identified proteins was highly correlated with TurboID expression (Fig. 2*C*). For each protein, linear analysis revealed a strong linear correlation between TurboID expression and protein intensity, with up to 97.0% of proteins showing a coefficient of determination greater than 0.5 (Fig. 2*D*). These results confirm that TurboID expression is highly correlated with the amount and signal intensity of enriched proteins, suggesting that expression levels of the TurboID-eGFP control may dramatically alter identified bait-protein interactomes.

As TurboID expression levels increased, two key trends emerged in the proteomics data. First, using protein abundances determined from unfractionated whole cell extracts, we determined that the proportions of proteins with different cellular abundances labeled and identified by varying TurboID-eGFP expression levels changed significantly (Fig. 2*E*). Proteins identified in the different TurboID-eGFP expressing cells were categorized into four quartiles according to their abundance (top 25%, 25–50%, 50–75%, and bottom 25%) (Fig. 2*F*). Notably, the number of identified low abundance proteins (bottom 25%) increased with increased TurboID-eGFP expression (Fig. 2*E*). To compare between TurboID-eGFP expression groups, the protein intensities from the lowest TurboID-eGFP expression group (eGFP1) were used as a baseline to calculate relative intensities in the other three groups. We first compared unnormalized protein intensities calculated using the sum of all unique peptide intensities (supplemental Fig. S2*A*) within protein abundance groups (Fig. 2*G*). For all protein abundance groups, there was a significant increase in relative mean protein intensity comparing cells with high TurboID-eGFP expression to those with low expression (eGFP4 *versus* eGFP1). This is expected given the observed correlation between TurboID-eGFP expression and total protein labeling (Fig. 2*C*). Further, comparing protein abundance groups showed a non-uniform increase in relative intensity with low abundance proteins showing a greater relative protein intensity increase (60.2-fold in bottom quartile proteins) compared to highly abundant protein (20.3-fold for top 1% abundant proteins) (Fig. 2*G*). Additionally, we compared the results using alternative protein intensity calculation methods, including Top1 and xTop (27) (supplemental Fig. S2, *A*–*C*). Despite differences in the mean values derived from the different protein abundance calculation methods, the overall trend remained consistent, with high-abundance proteins exhibiting smaller relative changes compared to low-abundance proteins. These results highlight potential issues when comparing identified proteins obtained from bait and control TurboID experiments with varying expression levels.

Data normalization approaches are routinely used to correct for differences in bait *versus* control protein expression. To test if data normalization would correct for differences observed comparing groups with different TurboID-eGFP expression levels, we used three normalization methods to analyze the patterns of relative intensity changes for proteins across the four TurboID-eGFP expression groups (supplemental Fig. S2*A*). In aggregate, all data normalization methods reduced the relative difference in protein intensities when comparing the eGFP1 expression group to the higher TurboID-eGFP expression groups (Fig. 2*H* and supplemental Fig. S2, *D* and *E*). However, differences in the effectiveness of these normalization methods for proteins with different cellular abundances were observed for all normalization methods. Total intensity normalization using R code and retention time-dependent normalization from DIA-NN(26) yielded highly similar results and performed the best on highly abundant proteins. These methods were less effective for low abundance proteins (Fig. 2*H* and supplemental Fig. S2*C*). In contrast, normalization using MaxLFQ produced nearly uniform changes in mean values across the four groups for proteins of different abundances (supplemental Fig. S2*E*). However, the highest and lowest abundance protein groups displayed clear de-enrichment when comparing TurboID-eGFP with different expression levels (supplemental Fig. S2*E*). Normalized data across the four TurboID-eGFP expression groups can significantly reduce protein variation when bait and control TurboID expression levels are not equivalent. However, these normalization methods are the most effective when the bait and control TurboID expression is the most similar. Even in those cases, differences in protein abundance classes persist.

In summary, these data demonstrate that significantly different eGFP interactomes would be captured and reported even when using the same protein as the bait and control group due to expression differences. Significant differences in TurboID expression between bait and control groups alter the proportion of enriched proteins with varying abundances and the extent of their intensity changes. These changes can lead to distorted data after normalization and alter the accuracy of differential analysis.

### Optimizing Control TurboID Expression for Effective Proximity-Based Interactome Studies

**Fig. 3:**
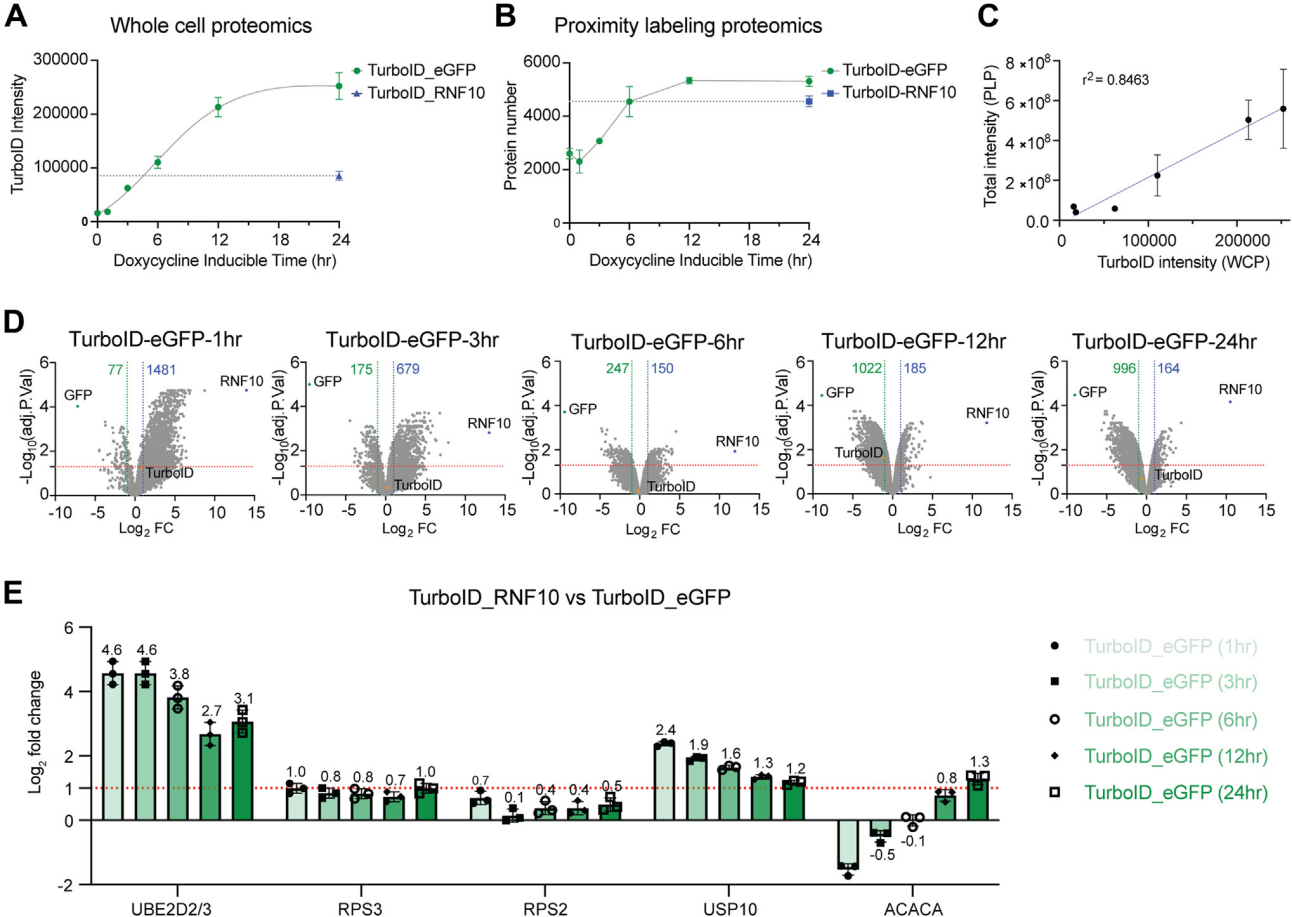
**Varying TurboID expression levels by induction time to match target protein expression**. *A*, HEK293 FlpIn cells with inducible expression of TurboID-eGFP were induced for 0, 1, 3, 6, 12, and 24 h. The abundance of TurboID was quantified by mass spectrometry from unfractionated cell extracts. TurboID-RNF10 expression was determined after 24 h of induction and is indicated by the blue dotted line. *B*, total number of identified proteins from streptavidin-enriched samples. *C*, correlation between TurboID expression obtained from whole-cell proteomics and total signal intensity of all identified proteins from streptavidin-enrichments. *D*, differential analysis between TurboID-RNF10 and five TurboID expression controls. *E*, log2-fold change of indicated proteins from streptavidin-enriched samples. Fold changes are determined by comparing Turbo-RNF10-expressing cell lines to five different control groups.

Our results demonstrate that inclusion of a TurboID expression control is essential to adequately monitor background labeling when performing proximity-based interactome studies. Further, our results indicate that the control TurboID expression level can significantly influence the intensities of identified proteins which can result in dramatically increased false positive identifications. Based on these results, we developed two cellular systems to try and match bait and control TurboID expression. We first used inducible expression systems for both the bait, TurboID-RNF10 and control, TurboID-eGFP. We established different control expression levels by varying the doxycycline induction time. As before, biotin incubation time remained consistent between all samples. As expected, increasing dox-induction time resulted in increased TurboID-eGFP expression levels (Fig. 3*A*). This elevation in TurboID expression resulted in a significant rise in both the number and total intensity of identified proteins (Fig. 3, *B* and *C*). A strong linear correlation was observed between TurboID expression and the total intensity of all identified proteins. We applied a total intensity normalization method at the peptide level to help comparative analysis between TurboID-eGFP and TurboID-RNF10.

Comparing the different doxycycline induction times of TurboID-eGFP with 24 h TurboID-RNF10 showed differing results with regards to the number of putative positive interacting proteins (Fig. 3*D* and supplemental Fig. S3). The number of identified RNF10 interacting proteins ranged from 150 to 1481 depending on which TurboID-eGFP expression control was used when using normalized data. As demonstrated earlier, high discordance between bait and control TurboID expression levels skewed results toward over enrichment when control TurboID expression was low compared to bait and under enrichment when control TurboID expression was high compared to bait expression. The 6 h TurboID-eGFP expression control most closely matched TurboID-RNF10 expression and resulted in the most normal distribution of enriched vs de-enriched proteins (Fig. 3*D*). RNF10 is known to interact with and ubiquitylate the 40S ribosomal proteins uS5 (RPS2) and uS3 (RPS3). RNF10 also interacts with the E2 enzyme, UBE2D2/3, and the deubiquitylating enzyme USP10 (19, 30, 31). Successful identification of ribosomal protein interactions is particularly challenging as ribosomal proteins are highly abundant and frequently identified as false positive background binding proteins. We specifically examined the relative fold change in protein intensity of these known RNF10-interacting proteins when comparing TurboID-RNF10 and each TurboID-eGFP control with different induction times. We also examined the endogenously biotinylated protein, ACACA, as a known negative control background protein. The fold-change variation trends of the five test proteins show noticeable diversity (Fig. 3*E*). The negative control protein ACACA showed a marked increase in fold change as control TurboID-eGFP expression increased relative to TurboID-RNF10, resulting in a false positive identification when the TurboID-eGFP with 24 h induction was used as the control. UBE2D2/3 consistently exhibited fold changes significantly greater than two and was relatively robust to differences in control vs bait expression (Fig. 3*E*). USP10 fold enrichment declined as control TurboID-eGFP expression increased. RPS3 and RPS2 displayed modest enrichment when using matched control TurboID-eGFP expression at 6 h and the enrichment increased as control expression increased (Fig. 3*E*). Taken together, using matched expression levels between bait and control proteins maximized identification of true positive interactions while minimizing false positive identifications.

**Fig. 4:**
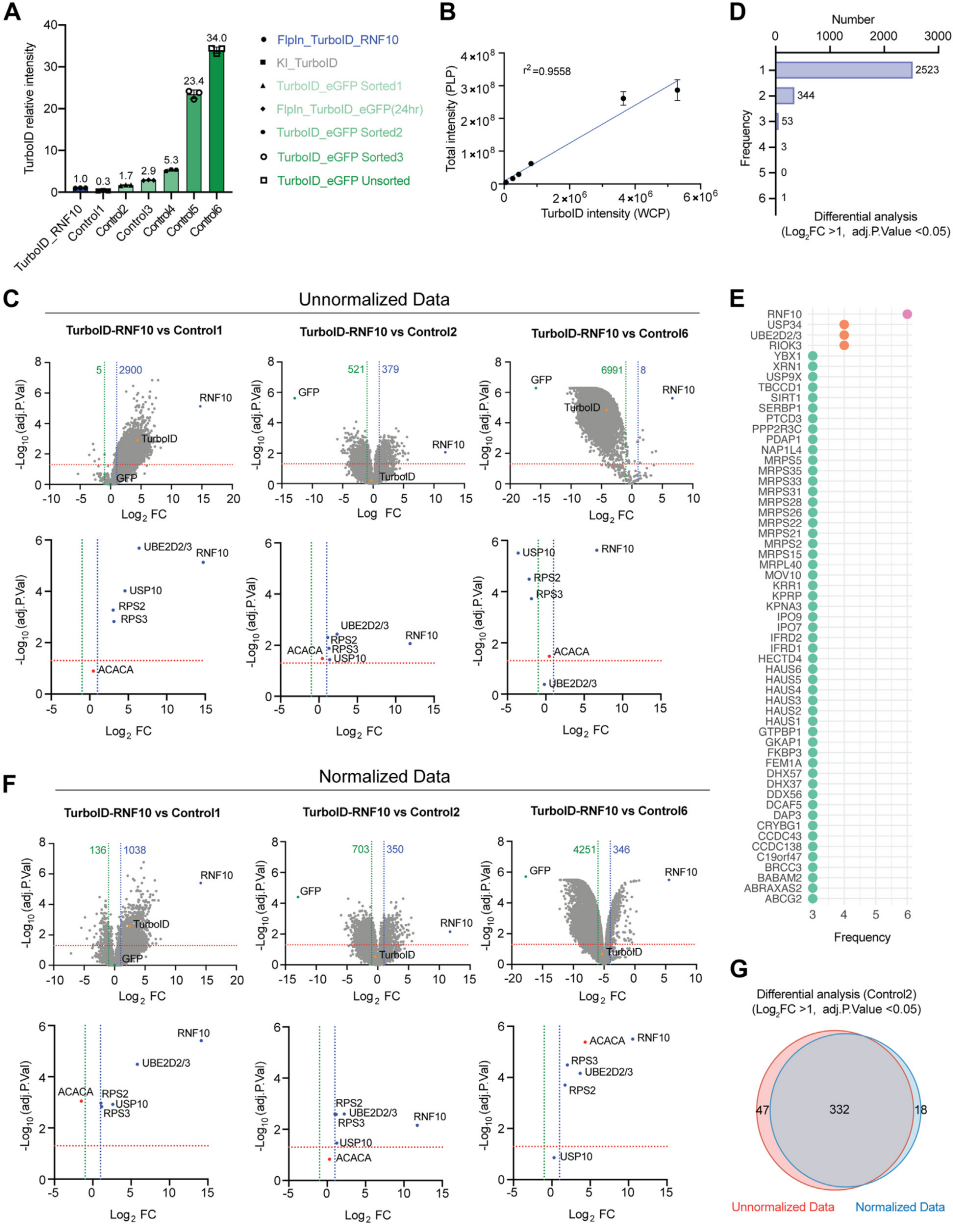
**TurboID expression-matched controls for RNF10 proximity labeling proteomics**. *A*, relative intensity of TurboID in the six controls compared with the TurboID-RNF10 group obtained through the FlpIn system with 24-h doxycycline induction. *B*, correlation between TurboID expression obtained from whole-cell proteomics and total signal intensity from streptavidin-enrichments for the six TurboID-eGFP control groups. *C*, differential analysis results of TurboID-RNF10 compared with three controls (Control1, Control2, Control6) based on non-normalized data. Four known RNF10-interacting proteins and the negative control ACACA are depicted. *D*, occurrence frequency of significantly enriched proteins obtained from differential analyses performed using the six controls. *E*, list of significantly enriched proteins with an occurrence frequency of three or more. *F*, differential analysis results of TurboID-RNF10 compared with three controls (Control1, Control2, and Control6) based on normalized data. *G*, Venn diagram comparing the differential analysis results obtained from non-normalized and normalized data using Control 2 as the control.

To validate the importance of TurboID expression-matched controls in differential analysis, we generated a larger set of TurboID-eGFP and TurboID-RNF10 expression systems using a combination of inducible and stable expression systems to obtain a broader range of both bait and control expression (Fig. 4*A* and supplemental Fig. S4*A*). Despite differences in generation methods, TurboID expression demonstrated a strong correlation with total intensity of all identified proteins (Fig. 4*B*). As expected, TurboID-RNF10 expressing cells had elevated RNF10 protein abundance, reaching 20.2-fold of the parental cell line (supplemental Fig. S4*B*).

Differential analysis using the highest expressing TurboID-RNF10 cell with the expression matched TurboID-eGFP control (control 2) revealed that the TurboID expression-matched control yielded significantly enriched proteins with the least proportion of background proteins (Fig. 4*C* and supplemental Fig. S4*C*). The results of the differential analysis using six different TurboID-eGFP controls were comprehensively evaluated, and the frequency of enriched proteins was quantified (Fig. 4*D*). Proteins with a high frequency of occurrence as being enriched with TurboID-RNF10 were considered high-confidence RNF10-interacting proteins (Fig. 4*E*). This analysis identified the known RNF10 interacting protein UBE2D2/3 and the iRQC factor RIOK3 as high-frequency interactors (32, 33, 34). When normalized data were used for differential analysis, the results showed that the enrichment of RNF10-interacting proteins was influenced by the controls but to a significantly lesser extent compared to the results obtained with non-normalized data. However, with increased TurboID expression in the controls, background proteins such as ACACA are identified as RNF10 interacting proteins after normalization (Fig. 4*F* and supplemental Fig. S4*D*). When the closest matched expression control, control2, was used, candidate interacting proteins obtained from non-normalized and normalized data were highly consistent (Fig. 4*G*). Consistently, ROC curve analysis using this control demonstrated strong discriminative performance across all comparisons (supplemental Fig. S4, *E* and *F*). Similar results were obtained when using two other TurboID-RNF10 expressing cell lines and their matched TurboID-eGFP expression lines (supplemental Fig. S4, *G*–*K*). These findings further illustrate that using bait expression-matched TurboID controls in differential analysis not only reduces background interference but also enhances the effectiveness and reliability of proteomics data normalization strategies.

### Evaluation of Expression Match Controls for the Identification of Ubiquitin Ligase Substrates

Reliable and robust proteomic methods are needed to rapidly identify ubiquitin ligase substrates. Our results with RNF10 indicate that using expression matched controls in proximity-labeling approaches can assist with identifying the known ribosomal RNF10 substrates. However, the highly abundant ribosomal proteins are somewhat atypical ubiquitin ligase substrates and ribosomal proteins are routinely identified in control samples which pose unique challenges for data analysis and normalization. To further evaluate our findings using expression-matched controls, we extended our evaluation to include HUWE1, a ubiquitin ligase with many reported substrates.

**Fig. 5:**
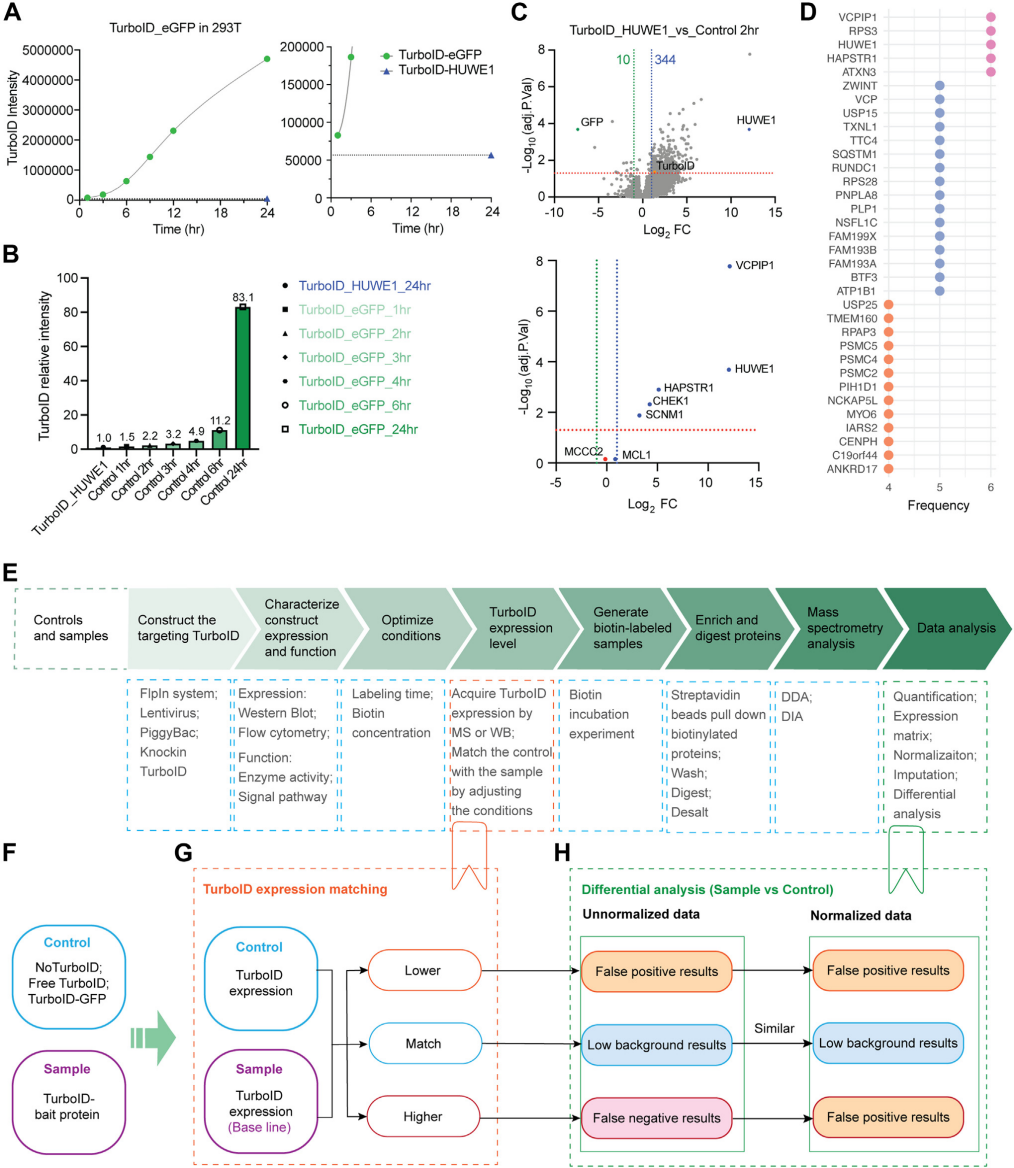
**Applying TurboID-matched controls to HUWE1 proximity labeling proteomics and workflow optimization**. *A*, TurboID expression in TurboID-eGFP and TurboID-HUWE1 groups. TurboID-eGFP controls were obtained using the PiggyBac system in HEK293T cells with different doxycycline induction times, while TurboID-HUWE1 samples were obtained with 24-h induction. *B*, relative intensity of TurboID expression in the six controls compared with the TurboID-HUWE1 group. *C*, differential analysis results of TurboID-HUWE1 compared to Control2h based on normalized data. *D*, List of significantly enriched proteins with an occurrence frequency of four or more in differential analysis using six controls based on the normalized data. *E*, recommended workflow for TurboID proximity labeling proteomics by incorporating a TurboID expression detection and matching step. *F*, different types of controls and samples. *G*, TurboID expression detection. *H*, differential analysis results under different TurboID expression matching conditions, including using non-normalized or normalized data.

We used a Piggybac-based recombination system to generate cell lines with inducible expression of TurboID-HUWE1 and the control TurboID-eGFP. We generated expression systems in both 293T and HAP1 cell lines to interrogate a larger potential HUWE1 substrate pool as some HUWE1 substrates are not expressed in all cell types. As before, control TurboID-eGFP expression was varied by altering the doxycycline induction time and, as expected, TurboID expression increased with dox-induction time (Fig. 5*A* and supplemental Fig. S5, *A* and *B*). TurboID-HUWE1 expression after 24 h of dox-induction was low compared to nearly all Turbo-eGFP controls making identification of matched expression samples difficult (Fig. 5*B* and supplemental Fig. S5*C*). Differential analysis of streptavidin-enriched proteins using multiple the TurboID-eGFP controls in both 293T and HAP1 cells confirmed that using TurboID expression-matched control effectively reduces background interference while increasing the identification of true interacting proteins (Fig. 5*C* and supplemental Fig. S5, *A* and *D*). In 293T cells, the 1-h dox-induction control was the most closely matched group, but differential analysis identified many background proteins. Even with the 2-h dox-induction control, the non-normalized data yielded a higher number of enriched proteins compared to the normalized results. The substantial difference in induction time between the control and sample may exacerbate the impact of background labeling using endogenous biotin sources. To address this issue, selecting bait-expressing populations with relatively higher TurboID levels, or sorting control cells to match expression levels, may help standardize expression induction durations and reduce background variability. Additional strategies, such as using weaker promoters or adjusting the inducer concentration, could further improve expression matching in future proximity labeling experiments. These results also suggest that varying TurboID protein expression and using multiple TurboID controls may be needed to confidently assign true interacting proteins using proximity-labeling approaches. Indeed, proteins that were frequently identified as putative HUWE1 interacting proteins using multiple TurboID controls were enriched for known HUWE1 interacting proteins, VCPIP1, VCP, ATXN3, as well as the HUWE1 substrates, HAPSTR1 and MCL1 (Fig. 5*D* and supplemental Fig. S5*E*). Collectively, our results establish an experimental workflow (Fig. 5, *E*–*H*) using appropriate expression-matched controls that can be employed to optimize protein interactome characterization using proximity-labeling proteomic approaches.

## Discussion

### Evaluating Strategies to Minimize False-Positive Identifications Using Proximity Labeling Proteomic Approaches

The selection of appropriate controls is critical for minimizing background protein interference and accurately identifying true positive interacting proteins using proximity labeling proteomics approaches. However, standardized guidelines for control selection and data analysis have not been established. This lack of standardization has resulted in the utilization of a wide variety of experimental controls (8, 9, 10, 11, 12, 13, 14, 15, 16, 17). Several strategies have been employed to reduce background interference, including adjusting bait protein expression levels, reducing sample input, optimizing biotin concentration, and altering biotin incubation time (8, 9, 12, 13, 35, 36). While application of these strategies, in isolation or combination, improves target interacting protein identification, it is often unclear what effect these strategies have on false-negative identifications. Often, it is difficult to interrogate published proximity-labeling interactome datasets due to the lack of appropriate controls and the high number of identifications that cannot be confidently classified as true-positive or false-positive interacting proteins. Thus, a systematic evaluation of appropriate controls for proximity-labeling approaches is needed to rigorously assess the sources of false-negative and false-positive identifications.

Numerous studies utilize samples that completely lack TurboID expression (TurboID-negative) to assess background binding to affinity matrices but completely overlook non-specific labeling by TurboID itself. Differential analysis using this experimental workflow cannot accurately differentiate true and false positive identifications. Other commonly used controls include the generation and utilization of samples expressing untagged TurboID or control-tagged TurboID (*e.g.* TurboID-eGFP) which serve to capture TurboID-mediated background labeling. These types of experimental controls provide significant advantages compared to TurboID-negative controls. However, differences in TurboID expression levels between the control and bait proteins directly influence differential analysis outcomes. When TurboID expression in the control is lower than in the samples, differential analysis based on both non-normalized and normalized data tends to overestimate differences, leading to increased false-positive identifications. Conversely, when control TurboID expression exceeds bait expression, differential analysis of non-normalized data may underestimate differences, resulting in false-negative identifications. Employing data normalization approaches can assist in correcting for differences in bait and control expression, but even normalized data can still result in abundant false-positive identifications. While normalization is useful for correcting technical variability, it may obscure biologically meaningful differences when TurboID expression levels between controls and samples are not properly matched. Therefore, TurboID expression-matched controls are essential for minimizing background interference and leveraging the benefits of normalization more effectively without excessively perturbing the data.

A recent study used a single expression vector with ribosomal skipping sequences to minimize differences in bait and control TurboID protein expression (20). This approach, similar to what we document here, allows for more robust assignment of false-negative background identifications. However, even when using coupled expression systems, differences in bait and control TurboID protein abundance were observed, complicating the assignment of false-positive interacting proteins. Additionally, this study noted that TurboID expression levels may influence the extent of background protein biotinylation. Using inducible systems allows for more control over bait and control protein expression. However, all these systems typically result in supra-physiological expression levels of bait proteins which may result in aberrant interactomes not observed in native conditions. Genome engineering approaches where coding sequences for proximity-labeling enzymes, like TurboID, can be introduced into the genome to generate fusion proteins expressed from the endogenous locus will be the preferred approach to minimize overexpression artifacts. Generating expression matched controls will be challenging for this approach, but having a selection of off-the-shelf cell lines with varying levels of control protein expression may be a suitable compromise.

An alternative strategy to reduce background in proximity labeling is the two-component BioID (2C-BioID) system (37), which separates the biotin ligase and bait protein and brings them together *via* chemically inducible dimerization. This design allows the uninduced state to serve as an internal control, minimizing background while preserving matched expression and localization. It also mitigates mislocalization caused by direct ligase fusions. The reduced background observed with 2C-BioID is consistent with our conclusion that matched TurboID expression between bait and control is a key factor for improving the specificity of proximity labeling experiments.

### Recommended Experimental Workflow

Our study reveals that false-negative identifications in TurboID experiments primarily originate from two sources: nonspecific adsorption of proteins to streptavidin beads and non-specific bait-TurboID protein biotinylation. Consequently, control samples should undergo identical sample processing steps to capture noise proteins bound to the beads. This study establishes that standard utilization of matched cell lines that express TurboID or TurboID-GFP fusion proteins is essential to account for TurboID-mediated background biotinylation. We describe a workflow to ensure that both major sources of background proteins are effectively accounted for and excluded during differential analysis. The resulting datasets will provide much more value to experimentalists that utilize interactome data as rationale for project development.

TurboID expression levels directly influence the composition and abundance of enriched proteins. Although data normalization can reduce intergroup variability, it inevitably introduces perturbations that compromise protein quantification accuracy. Generating samples with matched expression of control and bait-TurboID proteins minimizes background interference and enhances data processing robustness. These steps ensure consistency between normalized and non-normalized analyses. The matching strategy retains the advantages of normalization in correcting technical variations while mitigating its disruptive effects.

To accurately match TurboID expression between samples and controls, it is important that the bait and control proteins share the same subcellular localization. Otherwise, even with comparable overall expression in whole-cell lysates, differences in local TurboID abundance may arise. For baits confined to specific compartments, localization-matched controls such as TurboID-NLS or TurboID-NES fusions help ensure that observed differences reflect true proximity interactions rather than spatial bias.

Based on this study, we establish a standardized TurboID proximity labeling workflow which incorporates steps to measure control and bait TurboID expression while also selecting matched expression conditions (Fig. 5, *E*–*H*).

### Broadening the Application of Expression-Matched Controls

TurboID expression-matched controls can be generated through various strategies, such as inducible expression systems and fluorescence-activated sorting of TurboID-GFP fusion proteins based on fluorescence intensity. These strategies are broadly applicable across various cell types and experimental conditions, making them highly adaptable for different study designs. Mass spectrometry-based quantification of TurboID expression in whole-cell lysates can provide a robust and accurate method for selecting appropriate controls. Furthermore, these strategies facilitate the generation of a series of controls with varying TurboID expression levels. While this study focuses on the importance of expression-matched controls in TurboID-based proximity labeling proteomics, the same principle applies to other proximity labeling techniques, including the classic BioID system and more recently developed variants such as UltraID (6, 38). Similar concerns regarding appropriate control selection underscore the need to more broadly incorporate standardized controls when designing proximity labeling experiments. We focus on only two baits in this study, while proximity labeling approaches are often expanded to libraries of baits with hundreds of bait proteins interrogated in a single study. In these cases, having one-to-one matched expression controls for experimental baits and controls is not feasible. Here, using a small number of representative control samples with different expression levels (*e.g.*, low, medium, and high) to use as controls for baits in matched expression bins would be a reasonable compromise. This approach could reduce experimental complexity while accounting for systematic biases caused by expression variability among different baits, thereby facilitating better standardization in large-scale screens.

In summary, our study systematically evaluates key parameters that influence background interference and data interpretation in TurboID-based proximity labeling experiments. By establishing the necessity of TurboID expression-matched controls and proposing scalable strategies for their implementation, we provide a practical framework to enhance the specificity and reproducibility of proximity labeling proteomics. Our findings offer clear guidance for proximity labeling-based interactome studies and contribute to the broader goal of standardizing proximity labeling workflows for future applications.

## Data Availability

All the mass spectrometry proteomics data has been deposited to the ProteomeXchange (39) Consortium (https://www.proteomexchange.org/) *via* the PRIDE partner repository (40) with the dataset identifier (https://www.ebi.ac.uk/pride): PXD061540 (Token: gGM3LYvDICXw), PXD061541 (Token: W1OZpUsmHa9J), PXD061560 (Token: y04rJpaTyMTF), PXD061563 (Token: Fu7DqsL72GDg), PXD061564 (Token: tBiETREEi2VK), PXD061566 (Token: d2bNKCZLXjLq).

## Supplemental data

This article contains supplemental data.

## Conflict of interest

The authors declare the following financial interests/personal relationships which may be considered as potential competing interests: E. J. B. receives research funding from Pfizer Inc. All other authors have no competing financial interests to disclose.
