# Supplementary material for: Employing Expression-Matched Controls Enables High-Confidence Proximity-Based Interactome Classification
Source: Mol Cell Proteomics. 2025 May 27;24(7):101001. doi: 10.1016/j.mcpro.2025.101001 (PMC12226361; doi:10.1016/j.mcpro.2025.101001)
Supplement: Supplemental Data [file mmc1.docx]

**Employing Expression-Matched Controls Enables High Confidence Proximity-Based Interactome Classification**

Fulin Jiang^1^, Xuezhen Ge^1^, and Eric J. Bennett^1,2^

^1^School of Biological Sciences, Department of Cell and Developmental Biology, University of California, San Diego, La Jolla, CA 92093

^2^Correspondence: e1bennett@ucsd.edu

**Running title**: TurboID-matched control for proximity labeling proteomics

**Content**

**Supplementary Figures**:

Supplementary Figure S1 - Protein characterization and condition optimization in RNF10 proximity labeling proteomics experiments.

Supplementary Figure S2 - Data processing workflow and comparison of protein intensity variation using different data processing and normalization strategies.

Supplementary Figure S3 - Differential analysis between TurboID-RNF10 and five TurboID expression controls based on unnormalized data.

Supplementary Figure S4 - Comparison of RNF10 interactomes using different TurboID expression controls.

Supplementary Figure S5 - HUWE1 proximity labeling proteomics.

**Supplementary Tables**:

Supplementary Table S1 - Overview of datasets in this study. This table describes the samples used for comparative analysis and the type of sample (e.g. whole cell proteomics or biotin-enriched proximity proteomics) described in subsequent tables.

Supplementary Table S2 (Separate file) - Quantified proteins from six datasets, described in table S1. Each table describes the protein name, uniport accession, protein description, unique peptides matched, sequence coverage, and normalized protein intensities. The data normalization strategy is identified in the table tab. Annotated MS/MS spectra supporting the identification of UBE2D2/3 based on a single unique peptide in dataset 1 are included in a separate sheet.

Supplementary Table S3 - Variable isolation windows used for DIA data acquisition.

Supplementary Table S4 - List of described RNF10 interaction partners compiled from BioGRID, STRING, IntAct, and experimentally validated data.

**Source data:**

All mass spectrometry proteomics data have been deposited to the ProteomeXchange Consortium (https://www.proteomexchange.org/) via the PRIDE partner repository (dataset identifier at https://www.ebi.ac.uk/pride). The deposited files include protein identification and quantification results generated by DIA-NN and subsequent R-based analyses, together with annotated spectral library files in Skyline format. Reviewer account and password:

Dataset1: PXD061541 (Token: W1OZpUsmHa9J),

Dataset2: PXD061560 (Token: y04rJpaTyMTF),

Dataset3: PXD061563 (Token: Fu7DqsL72GDg),

Dataset4: PXD061566 (Token: d2bNKCZLXjLq),

Dataset5: PXD061564 (Token: tBiETREEi2VK),

Dataset6: PXD061540 (Token: gGM3LYvDICXw).

**
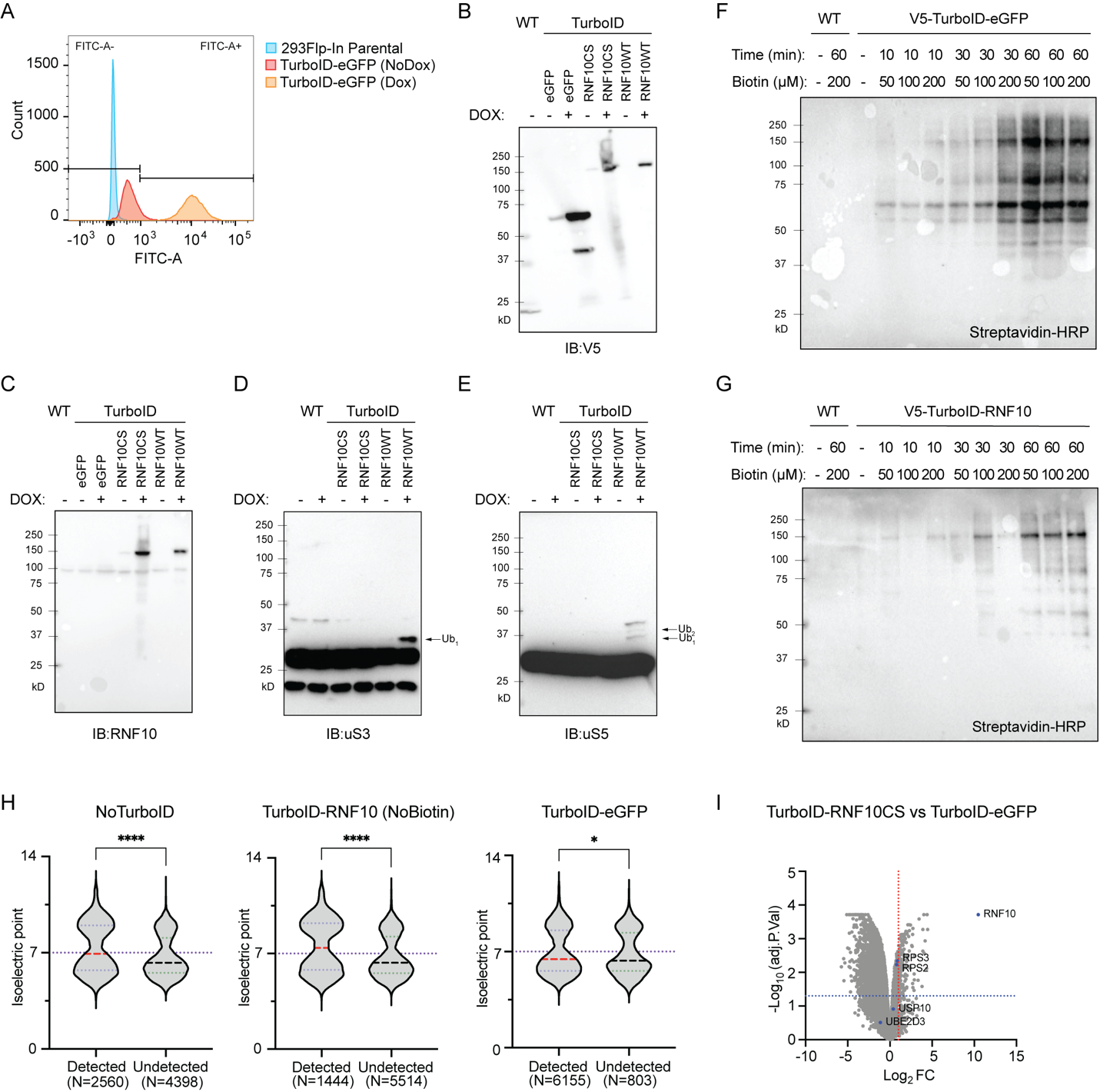
**

**Supplementary Figure S1 - Protein characterization and condition optimization in RNF10 proximity labeling proteomics experiments.**

A) Distribution of GFP intensities from the indicated cell lines and treatment conditions.

B-E) Whole cell extracts from cells expressing the indicated Turbo-ID fusion protein before and after doxycycline induction were analyzed by SDS-PAGE and immunoblotted as indicated. Arrows indicate ubiquitylated forms of uS3 or uS5.

F, G) HEK293 FlpIn cells with inducible TurboID-eGFP (F) or TurboID-RNF10 (G) expression were treated with doxycycline for 24 hours. Whole cell extracts were analyzed by SDS-PAGE and total biotinylated protein was detected using streptavidin-HRP. Cells were supplemented with biotin for the indicated times at the indicated concentrations.

H) Comparison of protein isoelectric points between streptavidin-enriched (detected) and unenriched (undetected) proteins for the indicated sample. Undetected proteins are those detected in unfractionated whole cell extracts but not detected in streptavidin-enriched samples. t test, *p < 0.05; ****p < 0.0001.

I) Volcano plot of differential analysis between TurboID-RNF10(C225S) and TurboID-eGFP.

**
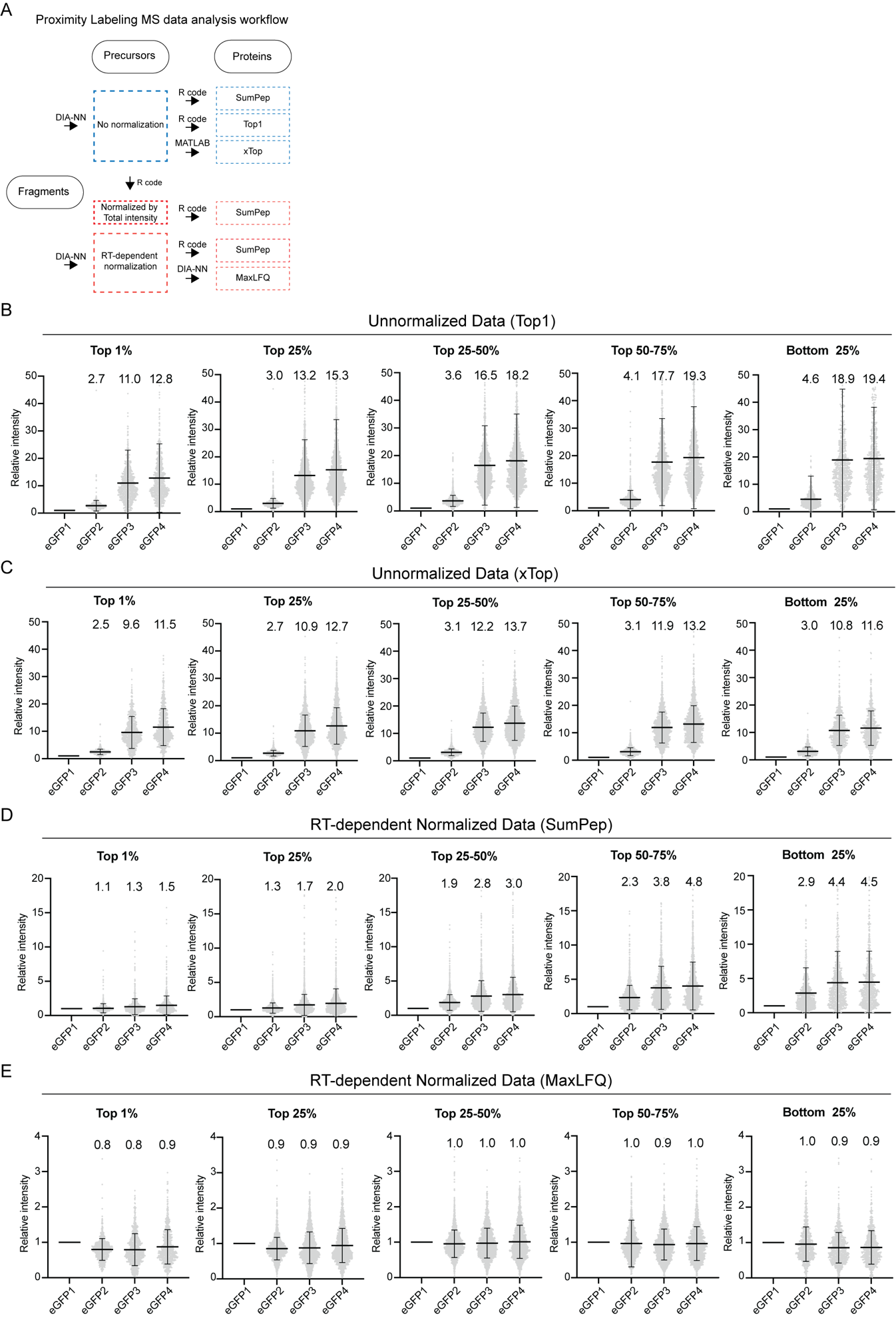
**

**Supplementary Figure S2 - Data processing workflow and comparison of protein intensity variation using different data processing and normalization strategies.**

A) Schematic of data processing workflow.

B-E) Relative intensity of streptavidin-enriched proteins from different protein abundance groups as compared to the intensity determined in the GFP-1 control group. Protein abundance groups were determined from proteomic analysis of unfractionated whole cell extracts. The number indicates the mean relative intensity. Unnormalized data using Top1 (B) or xTop (C) and normalized data based on retention time-normalized precursors using the Sumpep (D) or the MaxLFQ (E) are shown for comparison.

**
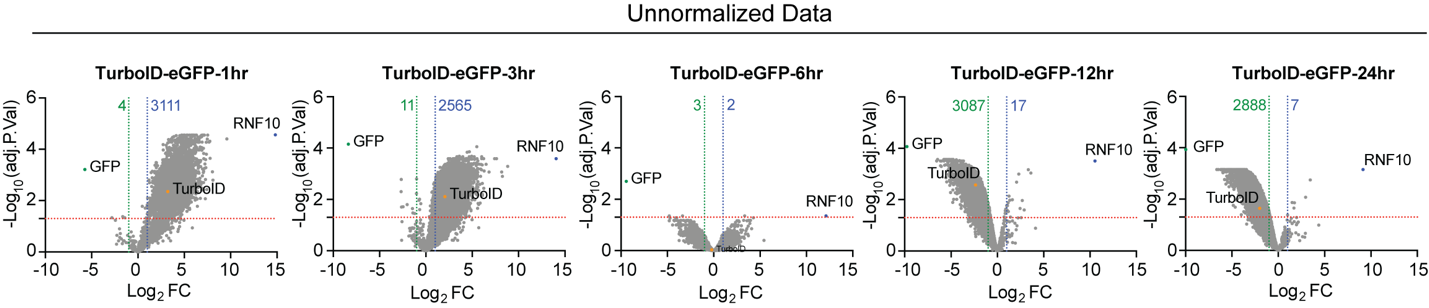
**

**Supplementary Figure S3 - Differential analysis between TurboID-RNF10 and five TurboID expression controls based on unnormalized data.** Differential analysis between TurboID-RNF10 and five TurboID expression controls without data normalization.

**
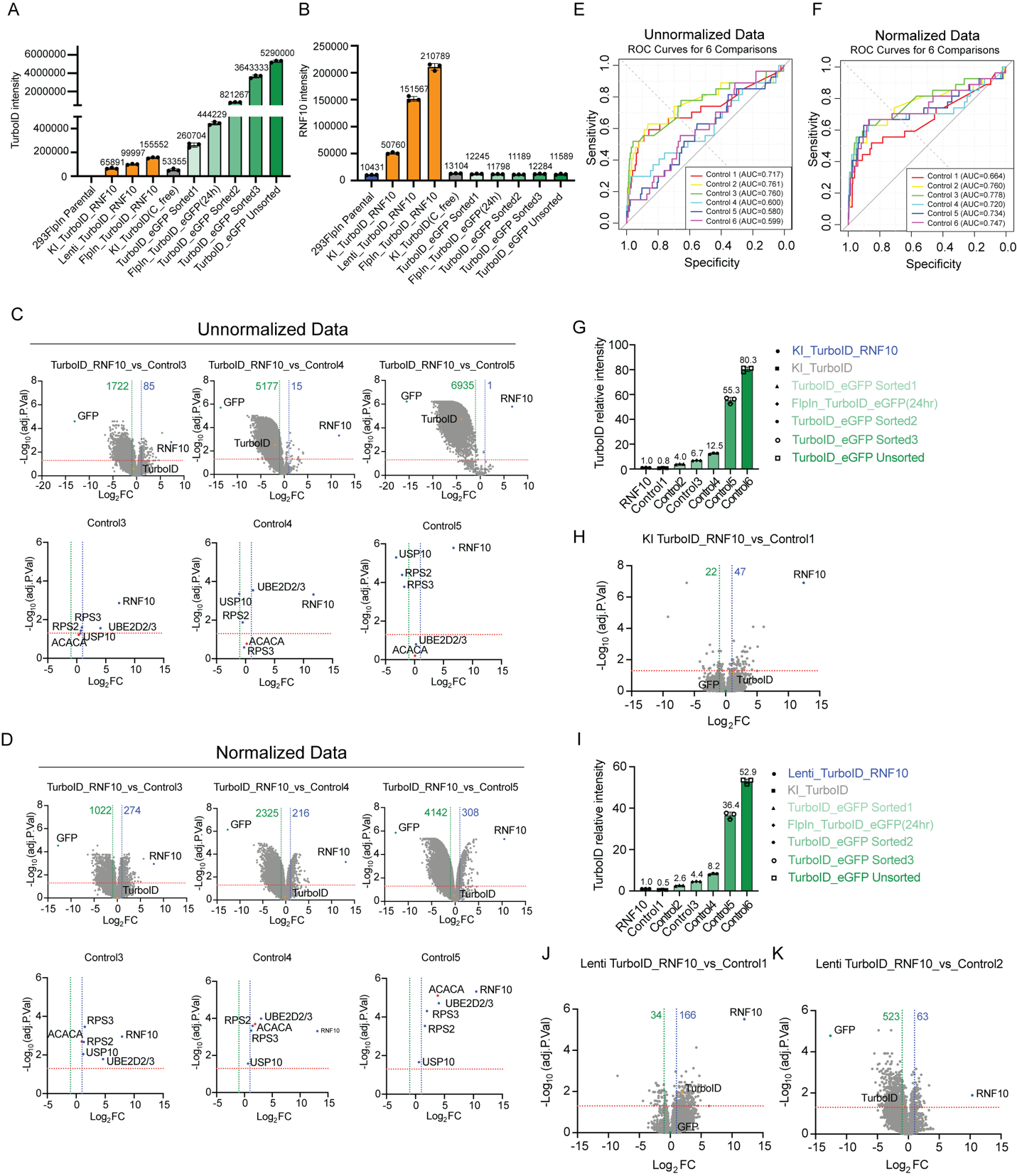
**

**Supplementary Figure S4 – Comparison of RNF10 interactomes using different varying TurboID expression controls.**

A) TurboID expression levels across three TurboID-RNF10 sample groups and six TurboID control groups.

B) RNF10 expression levels across three TurboID-RNF10 sample groups and six TurboID control groups.

C-D) Differential analysis of TurboID-RNF10 compared with three controls (Control3, Control4, Control5) based on unnormalized data (C) and normalized data (D).

E, F) ROC curve analysis of TurboID controls with varying expression levels, shown for unnormalized data (E) and normalized data (F).

G) Relative intensity of TurboID in the six controls compared with TurboID-RNF10 group obtained through the knock-in TurboID.

H) Differential analysis between knock-in TurboID-RNF10 and the matched TurboID expression control1 based on normalized data.

I) Relative intensity of TurboID in the six controls compared with TurboID-RNF10 group obtained through the lentiviral transduction.

J, K) Differential analysis between TurboID-RNF10 and two controls, Control1 (J) and Control2 (K) using normalized data.

**
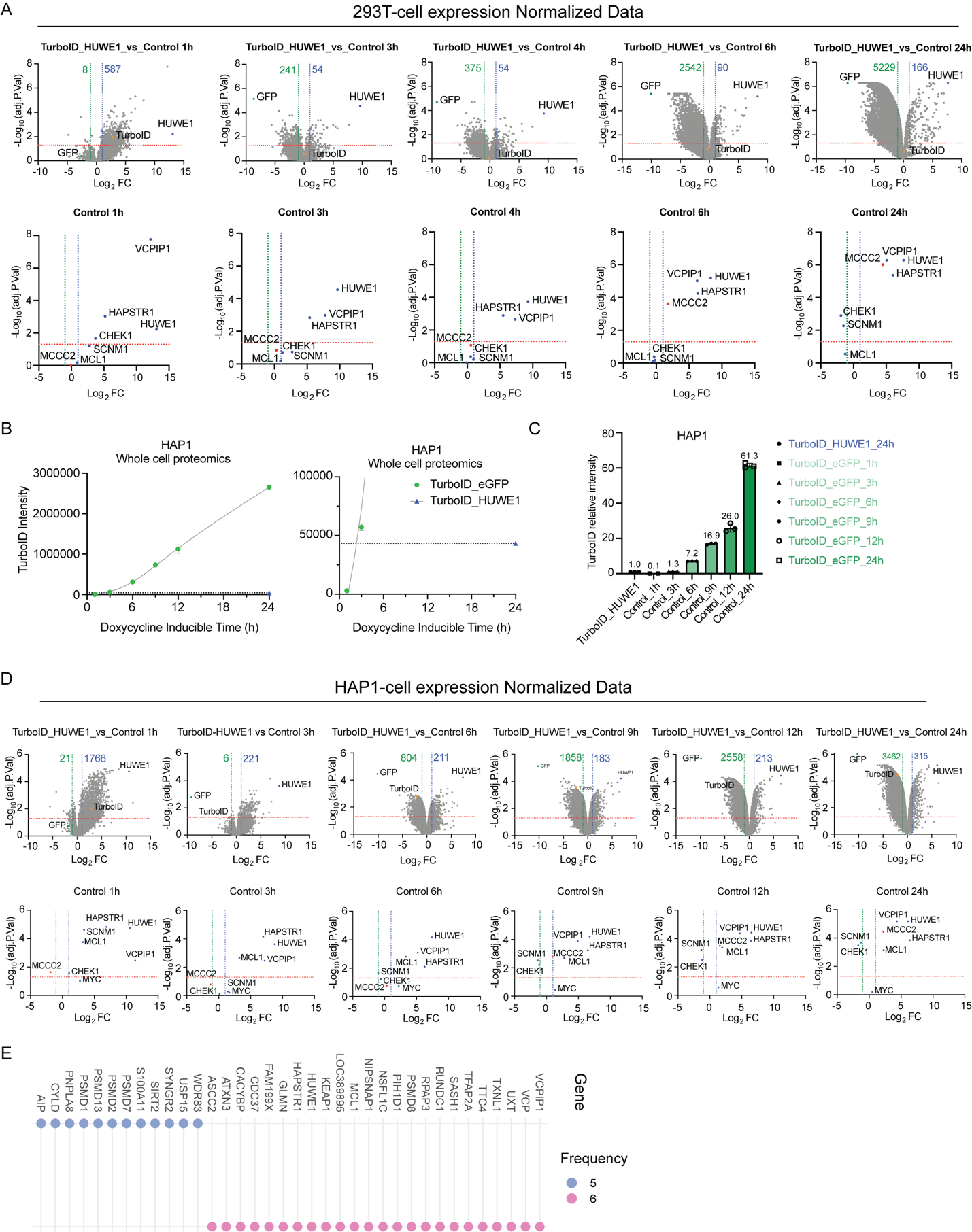
**

**Supplementary Figure S5 - HUWE1 proximity labeling proteomics.**

A) Differential analysis between TurboID-HUWE1 and various TurboID expression controls based on normalized data from HEK 293T cells.

B) TurboID expression in TurboID-eGFP and TurboID-HUWE1 groups in HAP1 cells. TurboID-eGFP controls were obtained using the PiggyBac system in HAP1 cells with doxycycline induction for 1, 3, 6, 9, 12, and 24 hours, while TurboID-HUWE1 samples were obtained with 24-hour induction.

C) Relative intensity of TurboID expression in the six controls compared with the TurboID-HUWE1 group.

D) Differential analysis between TurboID-HUWE1 and six TurboID expression controls based on normalized data.

E) List of significantly enriched proteins with an occurrence frequency of either 5 or 6.

**Supplementary Table S1** **- Overview of datasets in this study.** This table describes the samples used for comparative analysis and the type of sample (e.g. whole cell proteomics or biotin-enriched proximity proteomics) described in subsequent tables.

| **dataset** | **Sample type** | **Experiment** | **Description** |
| --- | --- | --- | --- |
| **i** | Proximity labeling proteomics | Control-type | 9 samples, three types of controls (Parental without TurboID, TurboID-RNF10 without Biotin, and TurboID-eGFP). |
|  |  | Lenti TurboID-eGFP expression | 12 samples, four TurboID-eGFP groups (Sorted1, Sorted2, Sorted3, and the unsorted group). HEK293 FlpIn cells. |
|  |  | TurboID-RNF10 expression | 12 samples, four groups (TurboID-RNF10 obtained via the Flp-IN transfection system, TurboID-RNF10 obtained via the lentiviral transfection system, TurboID-RNF10 obtained via knock-in, and the TurboID knock-in control). HEK293 FlpIn cells. |
| **ii** | Whole-cell extract proteomics | Control-type | 6 samples, two groups (Parental without TurboID and TurboID-eGFP). HEK293 FlpIn cells. |
|  |  | Lenti TurboID-eGFP expression | 12 samples, four TurboID-eGFP groups (Sorted1, Sorted2, Sorted3, and the unsorted group). HEK293 FlpIn cells. |
|  |  | TurboID-RNF10 expression | 12 samples, four groups (TurboID-RNF10 obtained via the Flp-IN transfection system, TurboID-RNF10 obtained via the lentiviral transfection system, TurboID-RNF10 obtained via knock-in, and the TurboID knock-in control). HEK293 FlpIn cells. |
| **iii** | Whole-cell extract proteomics | Flp-IN TurboID-RNF10 | 21 samples, seven groups (TurboID-eGFP induced with Dox for 0, 1, 3, 6, 12, and 24 hours via the Flp-IN transfection system, and the TurboID-RNF10 induced for 24 hours). HEK293 FlpIn cells. |
|  |  | TurboID-HUWE1 HAP1 cell line | 24 samples, eight groups (Parental without TurboID, TurboID-eGFP induced with Dox for 1, 3, 6, 9, 12, and 24 hours via the PiggyBac transfection system, and the TurboID-HUWE1 induced for 24 hours). HAP1 cells. |
| **iv** | Proximity labeling proteomics | Flp-IN TurboID-RNF10 | 21 samples, seven groups (TurboID-eGFP induced with Dox for 0, 1, 3, 6, 12, and 24 hours via the Flp-IN transfection system, and the TurboID-RNF10 group induced for 24 hours). HEK293 FlpIn cells. |
| **v** | Proximity labeling proteomics | TurboID-HUWE1 HAP1 cell line | 21 samples, seven groups (TurboID-eGFP induced with Dox for 1, 3, 6, 9, 12, and 24 hours via the PiggyBac transfection system, and the TurboID-HUWE1 induced for 24 hours). HAP1 cells. |
| **vi** | Proximity labeling proteomics | TurboID-HUWE1 HEK293T cell line | 21 samples, with seven groups (TurboID-eGFP groups induced with Dox for 1, 2, 3, 4, 6, and 24 hours via the PiggyBac transfection system, and the TurboID-HUWE1 group induced for 24 hours). HEK293T cells. |

**Supplementary Table S3 - Variable isolation windows used for DIA data acquisition.**

| MS Type | Cycle Id | Start Ion Mobility  [1/K0] | End Ion Mobility  [1/K0] | Start Mass  [m/z] | End Mass  [m/z] |
| --- | --- | --- | --- | --- | --- |
| MS1 | 0 | - | - | - | - |
| PASEF | 1 | 0.6 | 0.86 | 303.17 | 411.88 |
| PASEF | 1 | 0.86 | 1.6 | 697.37 | 708.83 |
| PASEF | 2 | 0.6 | 0.89 | 411.88 | 436.76 |
| PASEF | 2 | 0.89 | 1.6 | 708.83 | 720.32 |
| PASEF | 3 | 0.6 | 0.9 | 436.76 | 453.88 |
| PASEF | 3 | 0.9 | 1.6 | 720.32 | 731.72 |
| PASEF | 4 | 0.6 | 0.91 | 453.88 | 468.25 |
| PASEF | 4 | 0.91 | 1.6 | 731.72 | 743.67 |
| PASEF | 5 | 0.6 | 0.92 | 468.25 | 481.57 |
| PASEF | 5 | 0.92 | 1.6 | 743.67 | 756.91 |
| PASEF | 6 | 0.6 | 0.93 | 481.57 | 494.26 |
| PASEF | 6 | 0.93 | 1.6 | 756.91 | 769.9 |
| PASEF | 7 | 0.6 | 0.93 | 494.26 | 505.76 |
| PASEF | 7 | 0.93 | 1.6 | 769.9 | 783.39 |
| PASEF | 8 | 0.6 | 0.94 | 505.76 | 516.79 |
| PASEF | 8 | 0.94 | 1.6 | 783.39 | 797.39 |
| PASEF | 9 | 0.6 | 0.95 | 516.79 | 527.76 |
| PASEF | 9 | 0.95 | 1.6 | 797.39 | 811.89 |
| PASEF | 10 | 0.6 | 0.95 | 527.76 | 538.79 |
| PASEF | 10 | 0.95 | 1.6 | 811.89 | 826.41 |
| PASEF | 11 | 0.6 | 0.96 | 538.79 | 549.28 |
| PASEF | 11 | 0.96 | 1.6 | 826.41 | 841.42 |
| PASEF | 12 | 0.6 | 0.97 | 549.28 | 559.82 |
| PASEF | 12 | 0.97 | 1.6 | 841.42 | 856.77 |
| PASEF | 13 | 0.6 | 0.97 | 559.82 | 570.27 |
| PASEF | 13 | 0.97 | 1.6 | 856.77 | 873.13 |
| PASEF | 14 | 0.6 | 0.98 | 570.27 | 580.99 |
| PASEF | 14 | 0.98 | 1.6 | 873.13 | 889.91 |
| PASEF | 15 | 0.6 | 0.99 | 580.99 | 591.32 |
| PASEF | 15 | 0.99 | 1.6 | 889.91 | 908.44 |
| PASEF | 16 | 0.6 | 1 | 591.32 | 601.82 |
| PASEF | 16 | 1 | 1.6 | 908.44 | 926.46 |
| PASEF | 17 | 0.6 | 1 | 601.82 | 611.97 |
| PASEF | 17 | 1 | 1.6 | 926.46 | 947.44 |
| PASEF | 18 | 0.6 | 1.01 | 611.97 | 622.36 |
| PASEF | 18 | 1.01 | 1.6 | 947.44 | 968.48 |
| PASEF | 19 | 0.6 | 1.02 | 622.36 | 633.35 |
| PASEF | 19 | 1.02 | 1.6 | 968.48 | 990.95 |
| PASEF | 20 | 0.6 | 1.03 | 633.35 | 644.35 |
| PASEF | 20 | 1.03 | 1.6 | 990.95 | 1016.5 |
| PASEF | 21 | 0.6 | 1.04 | 644.35 | 654.64 |
| PASEF | 21 | 1.04 | 1.6 | 1016.5 | 1044.51 |
| PASEF | 22 | 0.6 | 1.05 | 654.64 | 665 |
| PASEF | 22 | 1.05 | 1.6 | 1044.51 | 1073.58 |
| PASEF | 23 | 0.6 | 1.06 | 665 | 675.79 |
| PASEF | 23 | 1.06 | 1.6 | 1073.58 | 1106.55 |
| PASEF | 24 | 0.6 | 1.08 | 675.79 | 686.32 |
| PASEF | 24 | 1.08 | 1.6 | 1106.55 | 1148.59 |
| PASEF | 25 | 0.6 | 1.09 | 686.32 | 697.37 |
| PASEF | 25 | 1.09 | 1.6 | 1148.59 | 1199.66 |

**Supplementary Table S4 - List of described RNF10 interaction partners compiled from BioGRID, STRING, IntAct, and experimentally validated data.**

| BioGRID  (Reported in at least two publications) | STRING  (Interaction score>0.5) | IntAct  (Confidence value>0.5) | Experiment |
| --- | --- | --- | --- |
| DDX23 | CMAS | HIP1 | RPS3 |
| IGF2BP3 | AKIRIN2 | WFS1 | RPS2 |
| KIAA1429 | UBXN6 | HSPB1 | RIOK3 |
| MEOX2 | UBE2D3 | HAUS8 | USP10 |
| MKRN3 | UBE2D2 | SPRED1 |  |
| MOV10 |  | TARDBP |  |
| RPS20 |  | CASP6 |  |
| TRIM26 |  | COQ8A |  |
| UBE2D1 |  | UBE2E3 |  |
| UBE2D2 |  | UBE2D2 |  |
| UBE2D3 |  | RNF11 |  |
| UBE2D4 |  | TRIM26 |  |
| UBE2E3 |  | TERF1 |  |
| UBE2O |  | HAUS5 |  |
| UTP14A |  | UBE2D3 |  |
| ZBTB48 |  | SNCA |  |
|  |  | UBE2D1 |  |
|  |  | UBE2D4 |  |
|  |  | HAUS6 |  |
|  |  | RAN |  |
